# An Algorithm for Treatment of Symptomatic Chronic Subdural Hematomas

**DOI:** 10.7759/cureus.56119

**Published:** 2024-03-13

**Authors:** Alice S Wang, Raphia Rahman, Arisa Ueno, Saman Farr, Jason Duong, Dan E Miulli

**Affiliations:** 1 Neurosurgery, Riverside University Health System Medical Center, Moreno Valley, USA; 2 Neurosurgery, California University of Science and Medicine, Colton, USA; 3 Neurosurgery, Arrowhead Regional Medical Center, Colton, USA

**Keywords:** middle meningeal artery embolization, tissue plasminogen activator (tpa), tpa, craniostomy, treatment algorithm, chronic subdural hematoma (csdh)

## Abstract

Introduction: Although chronic subdural hematoma (CSDH) is a common neurosurgical disease, there is a lack of algorithms for the treatment of asymptomatic and symptomatic CSDH. The purpose of this article is to describe an algorithm developed using our institutional experience for the treatment of symptomatic CSDH that aims to decrease symptoms and/or hematoma size or to completely resolve both. Our algorithm for treatment of symptomatic CSDH includes subdural drain (SDD) placement via twist-drill craniostomy (TDC) as the first-line treatment, followed by supplemental tissue plasminogen activator (tPA) as second-line treatment, with possible middle meningeal artery embolization (MMAE), followed by craniotomy as the last therapeutic option. This study investigated the efficacy of our institution’s algorithm in treating symptomatic CSDH.

Methods: A retrospective study was conducted from 2019 to 2023 identifying patients with CSDH treated with TDC. Electronic medical records were used to gather patient demographics, clinical presentation, radiographic findings, treatment modalities, and clinical outcomes.

Results: There were a total of 109 patients with 128 SDD placements. All 109 patients underwent TDC; among them, 37 patients received tPA instillation with three patients requiring craniotomy. Factors including age, gender, race, mechanism of injury, blood thinner usage, Glasgow Coma Scale (GCS), neurologic exam, thickness of CSDH, and midline shift were comparable for all patients regardless of treatment received. The mean number of neomembranes was higher in patients who eventually required craniotomy (4.5) compared to those treated with TDC only (1.8) and TDC+tPA (2.1) (p=0.0035). There was a greater mean hematoma drainage in patients who received tPA instillation without craniotomy (586.7 mL) than those treated with TDC only (293.0 mL) (p<0.0001). Clinical improvement was found in 52/72 patients (72.2%) treated with TDC only, 23/34 patients (67.6%) treated with TDC+tPA only, and 0/3 patients (0.0%) treated with TDC+tPA+craniotomy. Radiographic improvement in mean thickness of CSDH and midline shift, respectively, was found in patients treated with TDC only (p<0.0001; p<0.0001) and TDC+tPA (p<0.0001; p<0.0001) but not in TDC+tPA+craniotomy (p=0.1494; p=0.0762). There were also fewer neomembranes after TDC+tPA treatment only (2.1 vs. 0.5, p<0.0001). Seven patients were readmitted that did not follow the algorithm and only patients treated following the algorithm showed clinical and radiographic improvement.

Conclusions: Using our institutional algorithm, our study demonstrates successful clinical outcomes in treating symptomatic CSDH and recurrent CSDH with minimally invasive therapeutic interventions including SDD via TDC and tPA, thereby minimizing the utilization of more invasive interventions including craniotomy.

## Introduction

Chronic subdural hematoma (CSDH) is a common neurosurgical disease and is defined as liquefied hematoma in the subdural space that is at least seven days old [1]. The overall incidence of CSDH has been reported to be 1.7-20.6 per 100,000 persons per year [2]. Incidence rates have increased with age and the use of anti-coagulant and anti-platelet medications [2,3]. CSDH classically manifests following head trauma, and patients may recall a history of trauma [4]. A small CSDH may not cause any symptoms; however, its expansion may eventually exert a mass effect on nearby structures causing a variety of symptoms including headache, confusion, and limb weakness [2,5]. The diagnosis of CSDH is often made using a computed tomography (CT) scan or magnetic resonance imaging (MRI).

Currently, the most widely accepted theory underlying CSDH pathogenesis is that head trauma leads to the tearing of the bridging veins, which trespass through the dural border cell layer, resulting in a fluid collection in the subdural space [6,7]. In a CSDH, the outer membrane of the dural border cell layer has newly formed vasculature, which is suspectable to bleeding and contains tissue plasminogen activator (tPA), matrix metalloproteinases, and vascular endothelial growth factor that together create a self-perpetuating cycle of clotting, fibrinolysis, inflammation, angiogenesis, and recurrent hemorrhage [6-8].

There are no current existing guidelines for the treatment of asymptomatic patients with small CSDH collections. Literature has demonstrated the therapeutic option of reversing coagulopathy and starting a course of dexamethasone and atorvastatin followed by obtaining a repeat CT head at two weeks with serial imaging until resolution of hematoma or sooner if the patient becomes symptomatic [9-11].

However, there is a consensus that symptomatic patients should be treated surgically; therefore, surgical evacuation has become the mainstay of treatment. Three main surgical techniques are twist-drill craniostomy (TDC), burr-hole craniostomy (BHC), and craniotomy [11]. In TDC, local anesthesia is used and is often performed at the bedside [11]. The presence of neomembranes may interfere with maximal hematoma drainage and, therefore, additional instillation of tPA via subdural drain (SDD) can be successful in increasing CSDH drainage and reducing the incidence of recurrence without associated complications such as bleeding, meningitis, or ventriculitis [12,13]. In three meta-analyses studies comparing outcomes associated with these techniques, craniotomy was found to be associated with the least recurrence but highest rates of morbidity and mortality, whereas TDC was associated with greater recurrence but lower morbidity and mortality rates. BHC seems to provide a balance between efficacy and risks and is the most commonly performed technique [14-16]. To prevent recurrence, middle meningeal artery embolization (MMAE) can be performed to stop leakage of blood products and cause devascularization of permeable vascular membranes [17,18].

There is a lack of consensus on a standardized approach to treating symptomatic CSDH. Here, we describe our institution’s algorithm, which has evolved over the years based on our experience, that aims to decrease symptoms and/or hematoma size or to completely resolve both. At our institution, the first-line treatment for CSDH is TDC with the insertion of an SDD, which is effective in draining the hematoma as previously described in a cohort of 220 patients at our institution [19]. Supplemental intracatheter tPA may then be delivered into the subdural space if patients fail to improve clinically and/or radiographically to optimize hematoma drainage, as previously described in a cohort of six patients at our institution [13]. If there is no response to the less invasive therapies above, then patients undergo craniotomy. If at any stage patients show clinical improvement but with residual hematoma, they may additionally undergo MMAE. The authors provide a retrospective review regarding the efficacy and clinical outcomes in the treatment of symptomatic CSDH in 109 patients using our institutional algorithm.

## Materials and methods

This retrospective study conducted at Arrowhead Regional Medical Center from January 2019 to November 2023 received our Institutional Review Board approval. Informed consent was waived given this is a retrospective study. We retrospectively reviewed the efficacy and outcomes for patients with symptomatic CSDH treated using our institutional algorithm, which was developed based on our experience treating 225 patients from 2007 to 2018 [13,19]. Inclusion criteria included patients at least 18 years old and those who presented with symptomatic CSDH on initial presentation and were treated with TDC as the first-line treatment. Exclusion criteria included patients less than 18 years old, those with asymptomatic CSDH on initial presentation, and patients who underwent craniotomy for reasons that did not include treatment of CSDH. 

**Figure 1:**
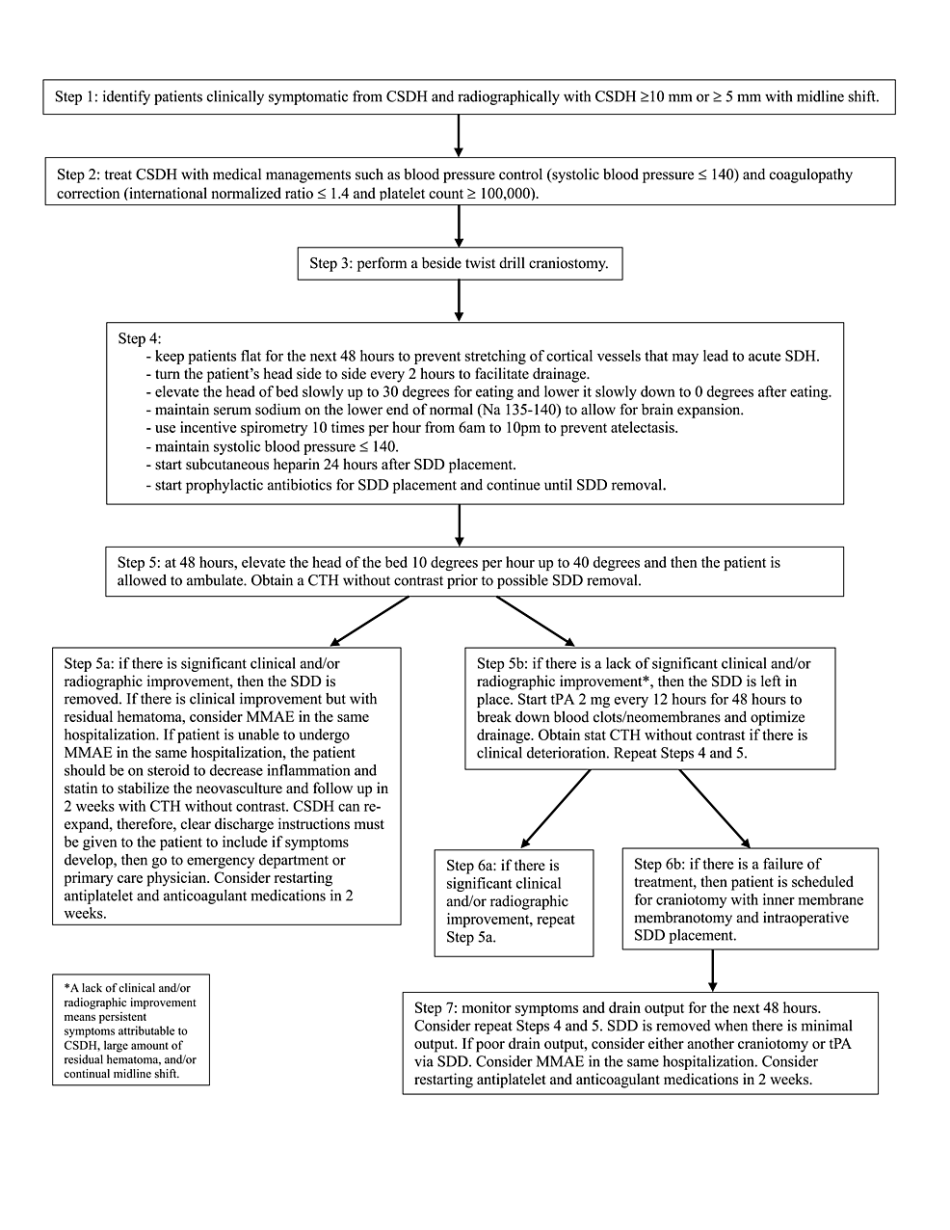
Our institution’s algorithm for symptomatic patients with CSDH CSDH, chronic subdural hematoma; SDD, subdural drain; CTH, computed tomography head; MMAE, middle meningeal artery embolization; tPA, tissue plasminogen activator The figure is the authors' own creation.

Patients were identified using Current Procedural Terminology (CPT) code 61108, which indicated they underwent TDC with an SDD placement with either a trauma catheter (Integra NeuroSciences TraumaCathTM Ventricular Catheter Set, REF Catalog Number INS8420 (Integra Lifesciences Corporation, Plainsboro Township, NJ, USA)) or a mushroom tip catheter (Integra NeuroSciences Subdural Drainage Catheter Kit, REF Catalog Number 951310 (Integra Lifesciences Corporation, Plainsboro Township, NJ, USA)) [19,20]. Either catheter is effective in hematoma drainage with similar complication risks as described in our experience with 205 patients from 2007 to 2017 [19]. Electronic medical records were reviewed to collect information regarding patient characteristics, clinical presentations, radiographic findings, treatment modalities, and clinical outcomes. The size of CSDH, amount of midline shift, laterality, and appearance were obtained from the radiology report; if the information was not provided in the report, CSDH was then measured using CT head (CTH) scans. The number of neomembranes was counted using CTH scans. The total amount of drainage was the sum of drainage output from the SDD via TDC. Statistical analyses including t-test, Fisher’s test, Chi-square test, One-way ANOVA, and Two-way ANOVA were performed using GraphPad Prism version 10.0.0 for Mac OS X (GraphPad Software, Boston, Massachusetts, USA) [21]. A p-value <0.05 is considered statistically significant. See Figure 1 for our institutional algorithm for the treatment of symptomatic CSDH. 

Twist-drill craniostomy 

A procedure timeout is performed to verify the correct patient, procedure type, and correct side of the procedure. Preprocedural antibiotics, anti-epileptic medications, and transfusion, if needed to correct coagulopathy, are given prior to the start of the procedure. Medications for conscious sedation are usually not administered, and local anesthetic, usually lidocaine with 1% epinephrine, is injected at the incisional site. The patients are placed supine and the surgical area is prepped in a sterile fashion followed by the marking of the incision site, which is premeasured using a CTH scan. A pilot hole is initially started using a handheld drill, then the trajectory of the drill is redirected obliquely. A durostomy is achieved using a sharp end of the tunnel metal rod. Then, an SDD trauma or mushroom-tip catheter is inserted along the longitudinal axis with a trajectory tangential to the skull. The tip of the catheter should be placed in the most gravity-dependent aspect of the head, for example, anterior to posterior. The subdural fluid collection is initially withdrawn slowly using a syringe connected to the implanted catheter. Then, the syringe is removed, and the implanted catheter is tunneled under the skin and connected to either a Jackson Pratt bulb (Cardinal Health, Dublin, OH, USA) or a Hemovac (One Medline Place, Mundelein, IL, USA) placed on full suction. A post-procedural CTH is used to verify SDD placement. See Appendix for craniotomy with inner membrane membranotomy. 

## Results

**Figure 2:**
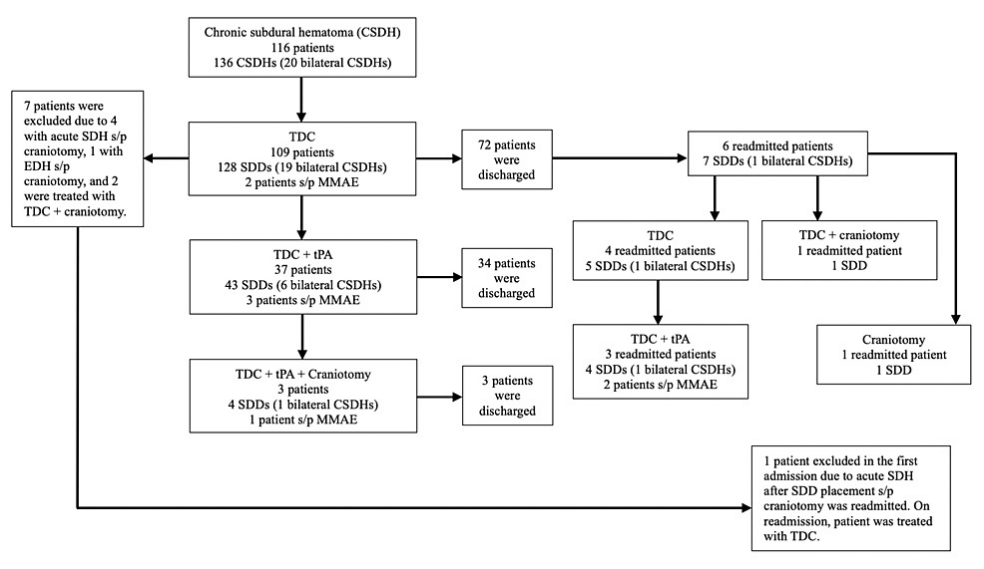
Treatment modalities for all 116 patients from January 2019 to November 2023 CSDH, chronic subdural hematoma; TDC, twist-drill craniostomy; SDD, subdural drain; s/p, status post; MMAE, middle meningeal artery embolization; SDH, subdural hematoma; EDH, epidural hematoma; tPA, tissue plasminogen activator The figure is the authors' own creation.

There were a total of 116 patients presenting with a total of 136 CSDHs (20 patients with bilateral CSDHs). Seven patients were excluded due to four patients with acute subdural hematomas needing emergent craniotomy (one patient had bilateral CSDHs), one patient with an epidural hematoma found on CTH prior to SDD removal needing emergent craniotomy, and two patients were treated with TDC followed by craniotomy. Among the 109 patients treated with TDC, 72 patients were discharged after TDC treatment alone, 37 patients required supplemental tPA with 34 of 37 patients discharged after TDC+tPA treatments, and three of 37 patients required further treatment with craniotomy. Among all the discharged patients, a total of six patients underwent MMAE in the same hospitalization (two patients from the TDC treatment alone, three patients from the TDC+tPA treatments, and one patient from the TDC+tPA+craniotomy treatments) (Figure 2).

To investigate factors that may underlie why some patients require additional treatments, we compared the patient characteristics and outcomes of the 109 patients treated with our algorithm (Table 1). The mean age was comparable among all the patients who received one or more treatment modalities. The majority of the patients were male and identified themselves as Caucasians or Hispanic. The most common chief complaint was headache. Ground-level fall was the most common mechanism of injury in patients who received TDC alone and TDC+tPA while most patients treated with TDC+tPA+craniotomy reported no trauma. The time of most recent trauma occurrence, if any recounted, ranged from the day of hospital presentation to six months prior to hospital presentation in patients treated with TDC alone and TDC+tPA, and was unknown in patients who eventually required craniotomy. Blood thinner usage was reported in 33.3% of the patients treated with TDC alone, 35.3% in TDC+tPA, and 100.0% in the TDC+tPA+craniotomy (p=0.5024). Physical exam findings (Glasgow Coma Scale (GCS) score and neurologic exam with motor dysfunction as the most common finding) and radiographic findings (CSDH laterality, appearance of CSDH, thickness of CSDH, and midline shift except a mean number of neomembranes) were comparable. The mean number of neomembranes was 1.8 in patients treated with TDC only, 2.1 in patients treated with TDC+tPA, and 4.5 in patients treated with TDC+tPA+craniotomy (p=0.0035) with a statistically significant difference between the TDC treatment cohort and TDC+tPA+craniotomy cohort (p=0.0025) and between the TDC+tPA cohort and the TDC+tPA+craniotomy cohort (p=0.0105). When comparing radiographic findings between patients not on blood thinners and those on blood thinners, there was no statistically significant difference in mean thickness of CSDH (18.6 mm vs. 19.0 mm, respectively, p=0.7029), mean midline shift (6.7 mm vs. 7.0 mm, respectively, p=0.7280), and a mean number of neomembranes (1.8 vs. 2.4, respectively, p=0.0852). Only drainage from SDD via TDC was used for analysis (drainage after craniotomy was not accounted for). The mean drainage was higher in patients who were treated with TDC+tPA than those treated with TDC only (586.7 mL vs. 293.0 mL, respectively, p<0.0001). 

**Table 1:** Patient characteristics and outcomes of the 109 patients treated with our institution’s algorithm The data has been represented as n, number of patients; %, percentage; mean±SD. *A p-value <0.05 is considered statistically significant. TDC, twist-drill craniostomy; tPA, tissue plasminogen activator; n, number of patients; SD, standard deviation; GCS, Glasgow Coma Scale; CSDH, chronic subdural hematoma; SDD, subdural drain; N/A, not available

Parameters		TDC (n=72 patients)	TDC+tPA (n=34 patients)	TDC+tPA+craniotomy (n=3 patients)	P-value
Age (years), mean±SD		69.7±15.6	66.1±16.6	74.0±14.1	0.4643
Sex, n (%)	Male	16 (22.2%)	8 (23.5%)	0 (0.0%)	>0.9999
	Female	56 (77.8%)	26 (76.5%)	3 (75.0%)	
Race, n (%)	Caucasian	38 (52.8%)	16 (47.1%)	1 (33.3%)	0.6541
	Hispanic	22 (30.6%)	11 (32.4%)	2 (66.7%)	
	Black	6 (8.3%)	5 (14.7%)	0 (0.0%)	
	Asian	2 (2.8%)	2 (5.9%)	0 (0.0%)	
	Others	4 (5.6%)	0 (0.0%)	0 (0.0%)	
Chief complaint, total number of findings (%)	Confusion	20 (14.5%)	9 (12.3%)	1 (20.0%)	N/A
	Headache	32 (23.2%)	19 (26.0%)	2 (40.0%)	
	Nausea/vomiting/dizziness	7 (5.1%)	5 (6.8%)	0 (0.0%)	
	Seizure	2 (1.4%)	2 (2.7%)	0 (0.0%)	
	Syncope	2 (1.4%)	3 (4.1%)	0 (0.0%)	
	Speech difficulty	6 (4.3%)	4 (5.5%)	0 (0.0%)	
	Fall	21 (15.2%)	11 (15.1%)	1 (20.0%)	
	Gait instability	21 (15.2%)	5 (6.8%)	0 (0.0%)	
	Weakness	22 (15.9%)	10 (13.7%)	1 (20.0%)	
	Numbness/tingling	5 (3.6%)	5 (6.8%)	0 (0.0%)	
Mechanism of injury, n (%)	Ground level fall	32 (44.4%)	15 (44.1%)	1 (33.3%)	0.9319
	Non-ground level fall	2 (2.8%)	0 (0.0%)	0 (0.0%)	
	Head strike	8 (11.1%)	3 (8.8%)	0 (0.0%)	
	Motor vehicle collusion	2 (2.8%)	2 (5.9%)	0 (0.0%)	
	Assault	3 (4.2%)	1 (2.9%)	0 (0.0%)	
	No trauma	22 (30.6%)	13 (38.2%)	2 (66.7%)	
	Unsure	3 (4.2%)	0 (0.0%)	0 (0.0%)	
Blood thinner usage, # (%)	Blood thinners	24 (33.3%)	12 (35.3%)	3 (100.0%)	0.5024
	No blood thinners	48 (66.7%)	22 (64.7%)	0 (0.0%)	
GCS on admission, mean±SD		13.8±2.1	14.1±1.8	14.0±1.7	0.7583
Neuro exam on admission, total number of findings (%)	Cognitive dysfunction	8 (12.5%)	6 (20.0%)	0 (0.0%)	0.7233
	Motor dysfunction	52 (81.3%)	23 (76.7%)	2 (100.0%)	
	Sensory dysfunction	4 (6.3%)	1 (3.3%)	0 (0.0%)	
Laterality of CSDH, total number of CSDH (%)	Left	46 (54.1%)	21 (53.8%)	3 (75.0%)	0.7895
	Right	39 (45.9%)	18 (46.2%)	1 (25.0%)	
Appearance of CSDH, total number of CSDH (%)	Homogeneous	21 (24.7%)	13 (33.3%)	0 (0.0%)	0.3652
	Heterogeneous	64 (75.3%)	26 (66.7%)	4 (100.0%)	
Thickness of CSDH on admission (mm), mean±SD		18.4±6.1	19.3±4.9	21.0±7.1	0.5104
Midline shift on admission (mm), mean±SD		6.5±4.9	7.6±5.2	5.1±3.2	0.3865
Number of neomembranes on admission, mean±SD		1.8±1.4	2.1±1.8	4.5±1.3	0.0035*
CSDH drainage via SDD placed in TDC, mean±SD		293.0±293.5	586.7±414.4	519.3±307.2	<0.0001*
GCS on discharge, mean±SD		14.2±1.9	14.2±2.5	13.3±2.1	0.7801
Neuro exam on discharge, total number of findings (%)	Cognitive dysfunction	5 (16.7%)	1 (7.7%)	0 (0.0%)	0.7836
	Motor dysfunction	23 (76.7%)	12 (92.3%)	2 (100.0%)	
	Sensory dysfunction	2 (6.7%)	0 (0.0%)	0 (0.0%)	
Overall neuro exam, n (%)	Improved	52 (72.2%)	23 (67.6%)	0 (0.0%)	<0.0001*
	Same	14 (19.4%)	9 (26.5%)	1 (33.3%)	
	Worse	3 (4.2%)	0 (0.0%)	2 (66.7%)	
	Expired	3 (4.2%)	2 (5.9%)	0 (0.0%)	
Thickness of CSDH on discharge (mm), mean±SD		8.7±4.2	8.0±4.0	9.8±4.7	0.5339
Midline shift on discharge (mm), mean±SD		2.9±2.5	2.8±2.6	1.2±1.1	0.4107
Number of neomembranes on discharge, mean±SD		1.1±1.0	0.5±0.6	N/A	0.0044*
Hospital length of stay (days), mean±SD		7.6±8.0	10.6±10.0	14.0±5.6	0.1347
Disposition, n (%)	Home	43 (59.7%)	22 (64.7%)	1 (33.3%)	0.2530
	Skilled nursing facility	16 (22.2%)	6 (17.6%)	1 (33.3%)	
	Rehabilitation	4 (5.6%)	2 (5.9%)	1 (33.3%)	
	Another hospital	5 (6.9%)	0 (0.0%)	0 (0.0%)	
	Expired	4 (5.6%)	2 (5.9%)	0 (0.0%)	
	Other	0 (0.0%)	2 (5.9%)	0 (0.0%)	

At the end of the hospital stay, mean GCS scores on discharge were comparable. Motor dysfunction remained the most common finding. Clinical improvement was found in 52/72 patients (72.2%) treated with TDC only, 23/34 patients (67.6%) treated with TDC+tPA only, and 0/3 patients (0.0%) treated with TDC+tPA+craniotomy. Radiographically, the mean thickness of CSDH and mean MLS were comparable. It was difficult to count the number of neomembranes after craniotomy; therefore, no data was available for analysis. The mean number of neomembranes in patients treated with TDC+tPA was significantly less than that in patients treated with TDC alone (0.5 and 1.1, respectively, p=0.0044). Hospital length of stay was comparable. Most of the patients treated with either TDC alone or TDC+tPA were discharged home while only one patient who eventually underwent craniotomy went home. 

Next, we compared the clinical and radiographic findings between admission and discharge for each treatment modality (Table 2). The mean GCS scores and neuro exam findings were comparable between admission and discharge for all patients treated with one or more treatment modalities. The mean thickness of CSDH and midline shift improved in patients treated with TDC alone and TDC+tPA. In patients treated with TDC+tPA, there was significantly fewer mean number of neomembranes upon discharge (2.10 vs. 0.593 p<0.0001).

**Table 2:** Comparison of clinical and radiographic findings between admission and discharge of the 109 patients treated with our institution’s algorithm The data has been represented as n, number of patients; %, percentage; mean±SD. *A P-value <0.05 is considered statistically significant. TDC, twist-drill craniostomy; n, number of patients; SDD, subdural drain; GCS, Glasgow Coma Scale; SD, standard deviation; CSDH, chronic subdural hematoma; tPA, tissue plasminogen activator; N/A, not available

TDC (n=72 patients, 85 SDDs)		Admission	Discharge	P-value
GCS, mean±SD		13.8±2.1	14.2±1.9	0.1082
Neuro exam, total number of findings (%)	Cognitive dysfunction	8 (12.5%)	5 (16.7%)	0.9144
	Motor dysfunction	52 (81.3%)	23 (76.7%)	
	Sensory dysfunction	4 (6.3%)	2 (6.7%)	
Thickness of CSDH (mm), mean±SD		18.4±6.1	8.7±4.2	<0.0001*
Midline shift (mm), mean±SD		6.5±4.9	2.9±2.5	<0.0001*
Number of neomembranes, mean±SD		1.8±1.4	1.1±1.0	0.0912
TDC+tPA (n=34 patients, 49 SDDs)		Admission	Discharge	P-value
GCS, mean±SD		14.1±1.8	14.2±2.5	0.9070
Neuro exam, total number of findings (%)	Cognitive dysfunction	6 (20.0%)	1 (7.7%)	0.5848
	Motor dysfunction	23 (76.7%)	12 (92.3%)	
	Sensory dysfunction	1 (3.3%)	0 (0.0%)	
Thickness of CSDH (mm), mean±SD		19.3±4.9	8.0±4.0	<0.0001*
Midline shift (mm), mean±SD		7.6±5.2	2.8±2.6	<0.0001*
Number of neomembranes, mean±SD		2.1±1.8	0.5±0.6	<0.0001*
TDC+tPA+craniotomy (n=3 patients, 4 SDDs)		Admission	Discharge	P-value
GCS, mean±SD		14.0±1.7	13.3±2.1	0.1835
Neuro exam, total number of findings (%)	Cognitive dysfunction	0 (0.0%)	0 (0.0%)	>0.9999
	Motor dysfunction	2 (100.0%)	2 (100.0%)	
	Sensory dysfunction	0 (0.0%)	0 (0.0%)	
Thickness of CSDH (mm), mean±SD		21.0±7.1	9.8±4.7	0.1494
Midline shift (mm), mean±SD		5.1±3.2	1.2±1.1	0.0762
Number of neomembranes, mean±SD		4.5±1.3	N/A	N/A

The overall complication rate was 12.46%. Procedural complications included 4/116 patients (3.45%) who developed acute SDH after SDD placement that required craniotomy, 1/116 patients (0.86%) who developed an EDH prior to SDD removal that required craniotomy, and 2/116 patients (1.72%) who were found to have SDD in the brain parenchyma due to placement of the twist-drill hole not made tangential to the skull on CTH that required SDD removal and replacement. Medical complications included 5/109 patients (4.59%) with aspiration pneumonia, 1/109 patients (0.92%) with deep vein thrombosis, and 1/109 patients (0.92%) with cardio arrest due to acute respiratory failure. The other 5/6 mortalities were due to unrelated causes. 

Readmissions

There were a total of seven readmitted patients with seven SDD placements. All seven readmitted patients except one patient who developed acute SDH after SDD placement requiring craniotomy were treated with TDC alone in their first admission. All seven patients were discharged home, returned to the emergency department (between six and 29 days after their first discharge), and readmitted for symptomatic CSDH. None of them underwent recommended MMAE in the same hospitalization or in an outpatient setting. None reported taking recommended steroids or statins. None reported taking blood thinners before readmission. Comparing the clinical and radiographic findings between the end of the first discharge and the beginning of the second admission, the mean thickness of CSDH was larger (10.2 vs. 17.0, p=0.0035) and the mean midline shift was greater (4.3 vs. 8.4, p=0.0383) while the mean GCS score (14.7 vs. 14.6, p=0.6036) and the mean number of neomembranes (2.0 vs. 2.4, p=0.5683) were comparable (Table 3). 

**Table 3:** Comparison of clinical and radiographic findings between first hospital discharge (n=7) and readmission of the seven readmitted patients (n=7) The data has been represented as n, number of patients and mean±SD. *A P-value <0.05 is considered statistically significant. n, number of patients; GCS, Glasgow Coma Scale; SD, standard deviation; CSDH, chronic subdural hematoma

Parameters	1st hospital discharge (n=7)	Readmission (n=7)	P-value
GCS, mean±SD	14.7±0.5	14.6±0.8	0.6036
Thickness of CSDH (mm), mean±SD	10.2±3.2	17.0±4.5	0.0035*
Midline shift (mm), mean±SD	4.3±1.3	8.4±5.0	0.0383*
Number of neomembranes, mean±SD	2.0±1.1	2.4±1.1	0.5683

Among the seven readmitted patients, four readmitted patients were treated using our algorithm (one patient was treated with TDC alone, and three patients were treated with TDC+tPA) with 2/4 patients undergoing MMAE in the same hospitalization. The other three readmitted patients did not follow the algorithm due to the surgeon’s preference (one patient was treated with TDC without supplemental tPA, one patient was treated with TDC+craniotomy, and one patient was treated with craniotomy) and none underwent MMAE (Figure 2). Similarly, we also looked at the patient characteristics and outcomes of the seven readmitted patients treated either following the algorithm or not (Table 4). Patients treated with the algorithm all showed clinical improvement with 3/4 patients discharged home. Those not treated with the algorithm did not improve, only 1/3 patients was discharged home.

**Table 4:** Patient characteristics and outcomes of the seven readmitted patients The data has been represented as n, number of patients; %, percentage; mean±SD. *A P-value <0.05 is considered statistically significant. n, number of patients; SD, standard deviation; GCS, Glasgow Coma Scale; CSDH, chronic subdural hematoma; SDD, subdural drain; N/A, not available

Parameters		Readmitted patients (n=4) treated with our institution’s algorithm	Readmitted patients (n=3) not treated with our institution’s algorithm	P-value
Age (years), mean±SD		62.3±18.9	73.7±7.4	0.3755
Sex, n (%)	Female	0 (0.0%)	1 (33.6%)	0.4286
	Male	4 (100.0%)	2 (66.7%)	
Race, n (%)	Caucasian	2 (50.0%)	2 (66.7%)	>0.9999
	Hispanic	1 (25.0%)	1 (33.6%)	
	Black	1 (25.0%)	0 (0.0%)	
Chief complaint, total number of findings (%)	Confusion	0 (0.0%)	2 (40.0%)	N/A
	Headache	2 (33.3%)	1 (20.0%)	
	Nausea/vomiting/dizziness	0 (0.0%)	1 (20.0%)	
	Seizure	1 (16.7%)	0 (0.0%)	
	Syncope	1 (16.7%)	0 (0.0%)	
	Speech difficulty	0 (0.0%)	0 (0.0%)	
	Fall	1 (16.7%)	0 (0.0%)	
	Gait instability	0 (0.0%)	0 (0.0%)	
	Weakness	0 (0.0%)	1 (20.0%)	
	Numbness/tingling	1 (16.7%)	0 (0.0%)	
Mechanism of injury, n (%)	Ground level fall	1 (25.0%)	0 (0.0%)	>0.9999
	No additional trauma	3 (75.0%)	3 (100.0%)	
Blood thinner usage, # (%)	Blood thinners	0 (0.0%)	0 (0.0%)	>0.9999
	No blood thinners	4 (100.0%)	3 (100.0%)	
GCS on admission, mean±SD		14.8±0.5	14.3±1.2	0.5385
Neuro exam on readmission, total number of findings (%)	Cognitive dysfunction	0 (0.0%)	0 (0.0%)	>0.9999
	Motor dysfunction	4 (80.0%)	2 (100.0%)	
	Sensory dysfunction	1 (20.0%)	0 (0.0%)	
Laterality of CSDH, total number of CSDH (%)	Left	2 (40.0%)	2 (66.7%)	>0.9999
	Right	3 (60.0%)	1 (33.3%)	
Appearance of CSDH, total number of CSDH (%)	Homogeneous	0 (0.0%)	0 (0.0%)	>0.9999
	Heterogeneous	5 (100.0%)	3 (100.0%)	
Thickness of CSDH on readmission (mm), mean±SD		19.2±3.7	13.3±3.1	0.0616
Midline shift on readmission (mm), mean±SD		9.8±4.9	6.2±5.1	0.3537
Number of neomembranes on readmission, mean±SD		2.4±1.1	N/A	N/A
CSDH drainage via SDD placed in TDC, mean±SD		362.6±214.9	101.0±50.9	0.1671
GCS on discharge, mean±SD		14.8±0.5	13.3±1.2	0.0749
Neuro exam on discharge, total number of findings (%)	Cognitive dysfunction	0 (0.0%)	1 (33.3%)	>0.9999
	Motor dysfunction	2 (100.0%)	2 (66.7%)	
	Sensory dysfunction	0 (0.0%)	0 (0.0%)	
Overall neuro exam, n (%)	Improved	4 (100.0%)	0 (0.0%)	0.0286*
	Worse	0 (0.0%)	3 (100.0%)	
Thickness of CSDH on 2nd discharge (mm), mean±SD		7.3±3.7	8.6±5.1	0.6914
Midline shift on 2nd discharge (mm), mean±SD		2.0±1.8	2.7±3.1	0.6792
Number of neomembranes on 2nd discharge, mean±SD		1.0±0.7	N/A	N/A
Hospital length of stay (days), mean±SD		12.5±11.0	16.7±6.4	0.5884
Disposition, n (%)	Home	3 (75.0%)	1 (33.3%)	0.4857
	Skilled nursing facility	1 (25.0%)	2 (66.7%)	

Next, we compared the clinical and radiographic findings between readmission and 2nd discharge (Table 5). Only the four patients who were treated with the algorithm in readmission showed radiographic improvement (CSDH thickness: 19.2 mm vs. 7.3 mm, respectively, p=0.0125; MLS: 9.8 mm vs. 2.0 mm, respectively, p=0.0223; and 2.4 vs. 1.0, respectively, p=0.0249). There were no procedural complications, medical complications, and mortality in all seven readmitted patients. None of these seven patients were readmitted a second time. 

**Table 5:** Comparison of clinical and radiographic findings between readmission and 2nd hospital discharge for readmitted patients The data has been represented as n, number of patients; %, percentage; mean±SD. *A P-value <0.05 is considered statistically significant. n, number of patients; GCS, Glasgow Coma Scale; SD, standard deviation; CSDH, chronic subdural hematoma; N/A, not available

Readmitted patients (n=4, 5 SDDs) treated with our institution’s algorithm		Readmission	2nd hospital discharge	P-value
GCS, mean±SD		14.8±0.5	14.8±0.5	>0.9999
Neuro exam, total number of findings (%)	Cognitive dysfunction	0 (0.0%)	0 (0.0%)	>0.9999
	Motor dysfunction	4 (80.0%)	2 (100.0%)	
	Sensory dysfunction	1 (20.0%)	0 (0.0%)	
Thickness of CSDH (mm), mean±SD		19.2±3.7	7.3±3.7	0.0125*
Midline shift (mm), mean±SD		9.8±4.9	2.0±1.8	0.0223*
Number of neomembranes, mean±SD		2.4±1.1	1.0±0.7	0.0249*
Readmitted patients (n=3, 2 SDDs) not treated with our institution’s algorithm		Readmission	2nd hospital discharge	P-value
GCS, mean±SD		14.3±1.2	13.3±1.2	0.3486
Neuro exam, total number of findings (%)	Cognitive dysfunction	0 (0.0%)	1 (33.3%)	>0.9999
	Motor dysfunction	2 (100.0%)	2 (66.7%)	
	Sensory dysfunction	0 (0.0%)	0 (0.0%)	
Thickness of CSDH (mm), mean±SD		13.3±3.1	8.6±5.1	0.2437
Midline shift (mm), mean±SD		6.2±5.1	2.7±3.1	0.1604
Number of neomembranes, mean±SD		N/A	N/A	N/A

## Discussion

Symptomatic CSDHs are treated surgically with TDC, BHC, or craniotomy, but there is no consensus on the first-line treatment modality [11]. A surgeon’s training, experience, and familiarity with each technique likely play a role in determining the surgical approach. For example, Santarius et al. reported that BHC was the most used technique at the University of Cambridge and they did not do TDC [5]. At our institution, TDC is the first-line treatment modality because it is the least invasive technique and does not require general anesthesia, which is important given the aging population, medical comorbidities, and increased blood thinner usage [2,3]. 

In our institution’s algorithm, we execute strict measures such as blood pressure control, coagulopathy correction, sodium control, flatbed rest with side-to-side turning, prophylactic blood thinner and antibiotics, and incentive spirometry to reduce the risks of complications associated with TDC. We recommend systolic blood pressure <140 and correcting coagulopathy to a goal of international normalized ratio <1.4 and platelet count >100,000 prior to TDC [22,23]. To facilitate brain expansion, we recommend maintaining sodium on the lower end of normal (Na: 135-140). Flatbed rest and gradual elevation of the bed are to prevent the stretching of cortical vessels that may lead to acute SDH. While early mobilization (on day 1) reduces the risk of medical complications when compared to delayed mobilization (after day 3), it may compromise the benefits of bed rest, which promotes brain expansion and prevention of tearing of fragile vessels [24]. We, therefore, decide on two days of bed rest and mobilize the patients before they have an increased risk of acquiring complications. Taking additional measures to decrease complications associated with immobility, we recommend starting subcutaneous heparin for chemical DVT prophylaxis 24 hours after SDD placement (a compromise between risk of bleeding and thrombosis), begin prophylactic antibiotics for prevention of drain-associated infection, and use of incentive spirometry for prevention of atelectasis. Complications were defined in the literature as residual hematoma that required reoperation, extradural hematoma that required operation, pneumonia, and death related to CSDH [25,26]. Our overall procedural and medical complication rate was 12.46%, which is within the range of previously reported complication rates of 11.25%-14% [25,26]. 

Based on our experience with 225 patients, we recommend leaving the SDD in for 48 hours to allow for brain expansion and maximum response time and decrease the risk of drain-related infection [5,13,19,27]. We also found from these 225 patients, there was no improvement without the requirement for further intervention if the SDD catheter remained greater than 48 hours [13,19]. The next step in our algorithm is the instillation of tPA to help increase hematoma drainage in patients who did not show significant clinical and/or radiographic improvement [12,13]. Our data align with the previous findings. If treatment fails with tPA, the next step is mini-craniotomy, which is the most invasive treatment modality and the last resort. Mini-craniotomy with fenestration of the inner membrane resulted in clinical and radiographic improvement and no symptomatic recurrences at the six-month follow-up [28]. Large craniotomy with extended membranectomy was associated with high morbidity and mortality and should be avoided [29]. With our understanding of the pathophysiology, we recommend inner membrane membranotomy under direct visualization to facilitate brain expansion, decrease stretching of the cortical vessels, and prevent SDH recurrences [6-8]. If there is still failure at this point, then another craniotomy or tPA administration after craniotomy can be considered [30,31]. None of the patients treated with the algorithm needed post-craniotomy tPA administration. 

The majority of the patients (106/109 patients, 97.2%) showed improvement and did not require more invasive craniotomy, demonstrating that our algorithm is effective in treating patients with symptomatic CSDH. As hypothesized, there were fewer neomembranes and more hematoma evacuation after tPA treatment in the patients treated with TDC+tPA only, which aligns with tPA’s role in fibrinolysis and hemolysis [6-8,13]. Previously, Brazdzionis et al. found blood thinner usage in 42.9% of the patients treated with TDC only and 66.7% treated with TDC+tPA [13]. Similarly, we found a trend of higher blood thinner usage in patients who eventually required additional treatments (33.3% treated with TDC only, 35.3% treated with TDC+tPA, and 100.0% treated with TDC+tPA+craniotomy). Although there were only three patients in the TDC+tPA+craniotomy group, it may be that the blood thinners make the hematoma resilient to TDC and the neomembranes resistant to tPA likely due to the outer membrane of the CSDH being prime to the blood thinners with unknown mechanisms, causing more bleeding, reabsorption, inflammation, and formation of neomembranes. In fact, we did find more neomembranes on initial CTH in patients who eventually required a craniotomy. The lack of statistically significant clinical and radiographic improvement after craniotomy may be due to the small sample size of three patients. All three patients were discharged and none of them returned for symptomatic recurrences, supporting the step for craniotomy in our algorithm. 

Although the hospital length of stay was not statistically significantly different in patients who received one or more treatment modalities, it should be noted that on average patients treated with TDC only were discharged in about one week, those with TDC+tPA in 1.5 weeks, and those with TDC+tPA+craniotomy in two weeks. Most of our patients were discharged home. 

Seven patients were readmitted because they did not follow our institution’s algorithm. They did not undergo prescribed MMAE and steroids and statins after their first hospitalization. Among them, only those who were treated following the algorithm showed clinical improvement, demonstrating the algorithm works regardless of whether they were previously treated. Therefore, the algorithm should be followed for all symptomatic patients with CSDH or recurrent CSDH. 

The recurrence rates after surgical intervention have been reported to be 12.2%-13.4% [32,33]. By embolizing the MMA, which provides the arterial supply to the neomembranes, it is thought to stop the self-perpetuating cycle of microbleeding and inflammation [34]. In our cohort, MMAE is considered for patients with clinical improvement but with residual hematoma. None of the 8/109 patients who received MMAE in the same hospitalization were re-admitted and the seven readmitted patients did not undergo MMAE, suggesting that MMAE is effective in managing recurrences. In fact, MMAE is emerging as a standalone technique [18,35,36]. Because using MMAE as a standalone is still in its infancy and we have yet to experience MMAE as a standalone treatment, in our algorithm, we consider MMAE after successful treatments with TDC, tPA, and craniotomy and recommend MMAE in the same hospitalization to avoid not following through with it in the outpatient setting. 

There are limitations to our study. This is a single-institution experience and is also a retrospective study. The management indications, biases, and deviations from other treatments may limit the generalizability of our algorithm. Neurosurgeons also have different levels of training and experience, which may affect their willingness to follow our institution’s algorithm. Moreover, most of the patients are lost to follow-up and, therefore, difficult to access the exact number of recurrences (defined as the number of readmissions). They may have presented to some outside hospital in which the electronic medical records were not available. Future studies should encourage patients to close follow-ups. 

## Conclusions

CSDH is a common neurosurgical pathology and yet there is a lack of standardized guidelines for treating symptomatic patients. Our institution has developed an algorithm to help standardize the approach for decreasing symptoms and size in treating CSDH. This study shows that our institution’s algorithm is successful in treating symptomatic CSDH and recurrent CSDH with good clinical outcomes for the majority of our study cohort and eliminates subjecting patients to a more invasive treatment modality such as craniotomy. This is important given the aging population and the increased use of anti-platelet and anti-coagulant therapies in patients who may not tolerate general anesthesia due to their medical comorbidities. We, therefore, highly encourage other institutions to use our institution’s algorithm in managing symptomatic patients with CSDH or recurrent CSDH to reduce or eliminate the symptoms and accumulation of blood. 

## Appendices

Methods

Operating room craniotomy with inner membrane membranotomy 

The patient is prepped in the standard neurosurgery fashion. The size of the craniotomy is dependent on the size and location of the chronic subdural hematoma (CSDH). After the dura opening, a red rubber catheter is used to irrigate all sides to remove the residual hematoma. The red rubber catheter remains in place. The outer membrane is observed and under direct visualization, and any bleeding from the outer membrane is stopped with bipolar cautery. Then, a small opening is made in the inner membrane (membranotomy) to insert the tip of the soft red rubber catheter, which is used to irrigate the space underneath the inner membrane. Due to the risk of inserting into the brain or injuring cortical vessels, the tip of the red rubber catheter is inserted only under direct visualization during irrigation, which allows a fluid-filled space to be created without touching the brain. While irrigating through the red rubber catheter, a subdural drain (SDD) is placed under direct visualization between the inner membrane and outer membrane and care is taken to not insert the SDD underneath the inner membrane that can cause cortical irritation or into the brain, which can cause cortical bleeding. Next, the inner membrane is now lifted off the brain by the irrigation fluid and a large membranotomy can now be safely performed under direct visualization. The SDD is left in place and the red rubber catheter is removed. The dura is approximated, and the SDD is tunneled greater than 5 cm away from the incision site and secured in place prior to the closure of the craniotomy. The SDD is connected to a Hemovac, which is then placed on suction. 

Bedside craniostomy with inner membrane membranotomy

Bedside craniostomy is considered an alternative to the operating room (OR) craniotomy for elderly patients with morbid medical comorbidities who likely will have a prolonged period of recovery if they have undergone general anesthesia. By performing this procedure under local anesthesia in our elderly population, we have noticed a decreased time to return to their baseline mental status, an overall better functional score, and a shorter duration in the intensive care unit (ICU) (unpublished). 

A procedure timeout is performed to verify the correct patient, procedure, and site of procedures. We recommend having the computed tomography head (CTH) scan in the patient’s ICU room, using navigation, maintaining systolic blood pressure <140, placing patient on nasal canal oxygen and monitoring pulse ox/end tidal carbon dioxide, and administering antibiotics prior to incision. For patient positioning, bring the patient’s head to the edge of the top of the bed. If indicated, may temporarily use soft bilateral wrist restraints to prevent the possibility of the patient reaching their head during the procedure. The hair at the surgical site should have been clipped from prior SDD placement; if needed, clip more hair to provide adequate exposure for tunneling a new drain laterally. For conscious sedation, we use morphine and versed. For regional nerve block at supraorbital and auricotemporal regions, we use 4.5 mL of 1% lidocaine with epinephrine (1:200,000), 4.5 mL of 0.25% marcaine, and 1 mL of 4.2% sodium bicarbonate. Then, take the previously inserted SDD off the suction. Prepare the procedure area with sterile betadine solution and remove the SDD and the staples from the original incision site. 

After drain and staple removal, prep the procedure area in standard neurosurgical fashion using 3MTM DuraPrepTMSurgical Solution (3M Health Care, St. Paul, Minnesota, USA), sterile blue OR towels, 3MTM IobanTM2 Anti-microbial Incise Drape (3M Health Care, St. Paul, Minnesota, USA), and a craniotomy drape [37,38]. Make a 3-4 cm incision over the original incision site and then extend the incision all the way down to the bone. Use a periosteal elevator to remove any pericranium from the skull and insert a small Weitlander retractor over the original burr hole. A Hudson brace hand drill (with a smaller drill bit) is used to carefully drill at the site of the original burr hole. Then switch to the larger drill bit (round and fat) to widen the original hole. Use a Kerrison rongeur to bite the lip off the edge of the burr hole in all directions. Place a 1x1 inch cottonoid soaked in lidocaine on the dural surface prior to opening for additional anesthetic. A #11 blade is used to open the dura in a cruciate fashion, followed by the use of portable electrocautery to burn back the leaflets. Gently bend a 12 Fraizer suction (with the stylet in place) into a soft C-curve (at least 60°) - take out the stylet - and direct only under direct vision into the burr hole and suction staying on top of the innermost membrane in order to protect the brain. Repeat this maneuver around the circumference (at 12 o’clock, at 3 o’clock, at 6 o’clock, at 9 o’clock) of the burr hole by taking out the suction and replacing it gently. Gently guide an 18 French red rubber catheter underneath the outer membrane using a small bayonet while it is connected to and irrigating from a large bulb irrigation and expect a return of chronic blood products after having opened the outer membranes. A medium Hemovac drain should be placed into the potential space along the superior aspect of the red rubber catheter and left in place. Tunnel the Hemovac out 5 cm laterally and secure in place with 2-0 nylon suture. Irrigate while slowly and carefully removing the red rubber catheter from the potential space while using a small bayonet to make sure the Hemovac stays in place. If any visible membranes are easily accessible repeat the procedure. Then, attention should be turned toward the innermost membrane. Pick up the inner membrane with Geralds with teeth and use a #11 blade to make a slight nick in the membrane while it is lifted off the surface of the brain. Then use a #4 Penfield to gain access to underneath the inner membrane. Gently guide only the tip of an 18F red rubber catheter underneath the inner membrane using a small bayonet while it is connected to and irrigating from a large bulb irrigator. Gently irrigate with bulb irrigation to create a space between the inner membrane and the surface of the brain. Slip a short dull nerve hook (tip always face up/superior) along the surface of the red rubber catheter and pull toward yourself to open the inner membrane only as much as you can see and have direct access to under the burr hole. Gently irrigate again with the bulb irrigation attached to the red rubber catheter. Use 2-0 vicryl to close the galea and then close the skin with staples. Place the Hemovac to suction. Perform a neuro exam and then send the patient for stat CTH to rule out any new intracranial hemorrhage. 
